# Polymorfism Of CYP2C9 And 3A5 and carbamazepine hypersensitivity reactions in Brazilian subjects

**DOI:** 10.1186/2045-7022-4-S3-P49

**Published:** 2014-07-18

**Authors:** Luciana Tanno, Daniel Kerr, Bernardo Santos, Leda Talib, Helcio Rodrigues, Wagner Gattaz, Jorge Kalil

**Affiliations:** 1 University of São Paulo, Clinical Immunology and Allergy Division, Brazil; 2 University of São Paulo, Laboratory of Neuroscience – LIM-27 Psychiatry, Brazil; 3 University of São Paulo, Nursing School, Brazil; 4 University of São Paulo, 3. Laboratory of Immunology – LIM-19 Clinical Immu, Brazil

## Background

The cytochrome P450 2C9 and 3A5 enzymes are predominantly found in the human liver, and have important functions in the metabolism of antiepileptics. Genetic polymorphisms in these genes are associated with altered enzymatic activities and may give rise to the risk of drug hypersensitivity. Therefore, we analyze the association between CBZ hypersensitivity reactions (CHR) and polymorphisms of CYP2C9 and 3A5 in a population of São Paulo, Brazil.

## Methods

Case-control study in which we genotyped the SNP of CYP2C9 (rs1057910, rs1799853) and 3A5 (rs776746) of samples obtained from 52 subjects with varying severities CHR, 82 tolerants, and 366 control subjects, all from São Paulo, Brazil. According to the alleles presente, each subject was classified as normal, decrease or increased funcion for the enzyme. The phenotype was evaluated based on standardized scoring systems using an adapted ENDA (European Network of Drug Allergy) questionnaire, medical records and on the clinical follow-up. The patch test with the culprit drug was performed according the ENDA recommendations.

## Results

We studied 500 subjects, 52 were validated as CHR, 28 Drug Reaction with DRESS and 14 SJS. Sixty-five percent of females and mean age was 43.6 years. Eighty percent had mixed ethinicity. Of all 41 drug patch tests, 28 (68%) were positive, in both SJS and DRESS. We found a strong association between normal activity of CYP3A5 and tolerants subjects when compared to CHR (p=0.0002 OR=4.8), but we observed homogeneous distribution of variants of CYP2C9 in our case and control groups. None of our groups presented positive association with CYP2C9 polymorphisms.

## Conclusion

Based on the association of the polymorphism of CYP3A5 and the tolerant group, we hypothesize that it can contribute to the tolerability of anti-epileptics. We could not find an association between CYP2C9 polymorphisms in any of these subjects, different from the previously described in different populations. Given the rarity of such reactions, more work is needed to translate observed differences in metabolism and pharmacokinetics into using genomic variation for predictive use.

